# Quantification of avian hazards to military aircraft and implications for wildlife management

**DOI:** 10.1371/journal.pone.0206599

**Published:** 2018-11-01

**Authors:** Morgan B. Pfeiffer, Bradley F. Blackwell, Travis L. DeVault

**Affiliations:** 1 USDA, APHIS, Wildlife Services, National Wildlife Research Center, Ohio Field Station, Sandusky, Ohio, United States of America; 2 School of Natural Resource Management, George Campus, Nelson Mandela University, George, South Africa; Institute of Animal Science, CZECH REPUBLIC

## Abstract

Collisions between birds and military aircraft are common and can have catastrophic effects. Knowledge of relative wildlife hazards to aircraft (the likelihood of aircraft damage when a species is struck) is needed before estimating wildlife strike risk (combined frequency and severity component) at military airfields. Despite annual reviews of wildlife strike trends with civil aviation since the 1990s, little is known about wildlife strike trends for military aircraft. We hypothesized that species relative hazard scores would correlate positively with aircraft type and avian body mass. Only strike records identified to species that occurred within the U.S. (*n* = 36,979) and involved United States Navy or United States Air Force aircraft were used to calculate relative hazard scores. The most hazardous species to military aircraft was the snow goose (*Anser caerulescens*), followed by the common loon (*Gavia immer*), and a tie between Canada goose (*Branta canadensis*) and black vulture (*Coragyps atratus*). We found an association between avian body mass and relative hazard score (*r*^*2*^ = 0.76) for all military airframes. In general, relative hazard scores per species were higher for military than civil airframes. An important consideration is that hazard scores can vary depending on aircraft type. We found that avian body mass affected the probability of damage differentially per airframe. In the development of an airfield wildlife management plan, and absent estimates of species strike risk, airport wildlife biologists should prioritize management of species with high relative hazard scores.

## Introduction

One of the most ubiquitous human-wildlife conflicts involves wildlife colliding with transportation [1]. These collisions usually result in the death of the animal, damage to the vehicle, and even injuries to humans or human fatalities [2]. Compared to other countries, the United States of America (U.S.) has the most reported wildlife collisions (strikes) with aircraft, likely because of numerous aircraft operations, mandatory strike reporting in some sectors, and an abundance of large-bodied birds [3, 4]. Ranking of wildlife hazards to civil aircraft has been conducted regularly since the 1990s [5–7], but rarely for military aviation. Furthermore, to our knowledge, relative hazard scores (RHS) for military aircraft have not been calculated or published [8]. According to the United States Air Force (USAF) wildlife strike database from 1985–1998, an average of $35 million (U.S.) in damage was attributed to wildlife strikes annually [8]. To mitigate this risk of wildlife strikes, most United States military bases with a flying mission require a Bird/Wildlife/Animal Aircraft Strike Hazard program [9,10].

A necessary component of estimating strike risk (see below), and thus prioritizing resources and time for mitigating wildlife strikes, is the ranking of wildlife by the hazard they pose to aviation. Hazard is defined in terms of the likelihood of damage, substantial damage, or negative effect-on-flight procedures when a strike event occurs [11]. This hazard, or severity, of a strike with an animal is one component used to estimate strike risk, along with a measure of the frequency of the event (i.e. how often strikes occur per species) [12]. Because RHS have never been calculated for military aircraft, our objective was to calculate these scores as the first step in estimating risk, which is dependent upon a number of local-level variables [12], in addition to mission type.

Wildlife hazard ranking has been conducted for civil aviation in a number of ways [6, 11, 13]. A previous study on USAF wildlife strikes ranked species groups (i.e. species that are closely related phylogenetically) based on the number of strikes within each damage class (i.e. categories of monetary damage costs to aircraft) and found that vultures (Cathartidae), geese (Anatidae), pelicans (Pelecanidae), blackbirds/starlings (Icteridae/Sturnidae), and buteo hawks (Accipitridae) were ranked as most hazardous, in that order [8]. However, not all wildlife strikes result in aircraft damage; therefore, damaging or negative effect-on-flight strikes per species should be considered as a proportion of all strikes (including non-damaging strikes) for that species or group [11]. In recent wildlife hazard rankings for civil aviation, a composite hazard score was calculated that included the sum of the ranks of strikes with damage, substantial damage, and strikes that had a negative effect-on-flight [11, 12, 14]. Replicating the methods of the previous examples must be done with special consideration of the differences between civil and military aviation and the assumed variation in the bird strike risk.

Military and civil operations differ in their mission types, including air speeds and maneuverability, which could affect wildlife hazard scores. During certain training procedures, military aircraft travel at high speeds and close to the ground, which likely increases the strike hazard compared to civil aircraft because of more time spent in bird rich altitudes [15]. Damage to aircraft is a function of kinetic energy which is the product of the mass of the bird in relation to the velocity of the aircraft [16]. However, birds with smaller body mass might still pose a substantial hazard to military, as compared to civil aircraft because of the increased aircraft speeds, particularly at lower altitudes, characteristic of military operations. In other words, whereas civil strikes involving smaller birds generally pose less hazard, collisions of small bird species with military aircraft can involve much higher airspeeds. Hence, a hazard difference between civil and military aviation may be more noticeable with the smaller rather than the larger birds. In addition, unlike civil aviation, military training operations can generally adjust their flight schedule if hazards, including the risk of bird collisions, is predicted to be elevated [17].

Given these considerations, our purpose was to calculate avian RHS for the United States Navy (USN), including the United States Marine Corp, and the USAF. We predicted that aircraft type and body mass would influence RHS [11]. Because military airframes are designed for a variety of purposes and generally only conduct certain maneuvers (e.g. fighter airframes that accelerate rapidly and turn with high precision vs. cargo airframes designed to move heavy payloads), we calculated RHS separately for grouped military airframe type. We predicted that airframes with more sensitive components would experience higher RHS compared to robust airframes. Calculating hazard scores in this way can help prioritize species-specific wildlife management plans for military airfields across the U.S. serving different mission types. Based on previous work involving civil aircraft [6, 11], we predicted that body mass and migration flyway (Central, Mississippi, Pacific, Atlantic) would contribute to predicting level of damage (see below) for military aircraft. Lastly, because of differing reporting rates, we predicted that probability of damage would differ between the two military branches. Furthermore, significant relationships between body mass and RHS could be used to calculate hazard scores for species that are not currently in our dataset (e.g. solve the equation for RHS based on avian body mass).

## Materials and methods

We used wildlife strike records from databases maintained by the USN and the USAF. The USN wildlife strike database spanned 27 years (1990–2017) and contained 21,661 wildlife strike records. The USAF dataset spanned 23 years (1994–2017) and contained 104,129 wildlife strike records. Wildlife remains found on an aircraft after a strike event are required to be sent to the Feather Identification Lab at the Smithsonian Institution, where personnel identify wildlife based on feather characteristics and/or DNA [18]. Some species information for the USN database was gleaned from the Smithsonian Institution’s internal database by matching the event number.

We filtered these databases to only include strikes that occurred within the U.S. where the species involved in the strike was identified. We did not filter strikes by altitude (i.e. < 152 m), which was done in similar studies with civil aviation to focus on the airport environment [11]. Military aircraft do not adhere to the 3° glide slope of ascent and decent of civil aircraft, from which the threshold of 152 m is based [19], and routinely conduct low-level flights. If multiple animals were involved in one strike event, they are entered as separate strikes, but with the same report number. To prevent duplication, only one strike per event was used (i.e. duplicate report numbers were removed). Duplicate report numbers were removed based on body size; the larger of the two species was retained. Less than 12% of all records involved more than one reported species, in which we kept the larger of the two species for the analysis. Only species with more than 20 strikes were used in our analyses [12, 14]. To reduce complexity and facilitate management decisions, those species involved in fewer than 50 strikes were combined into species groups (S1 Table) based on phylogeny [20]. Species groups (*n* = 17) were composed of closely related species as per [20] and did not include any strikes in which guild was identified but not species. A total of 12 species with more than 20 but less than 50 strikes (noted in S1 Table) could not be grouped because they were not closely related to other species and left as is in the analyses A similar species grouping scheme is accepted by the FAA in the management of hazardous species at airports and this scheme allows us to compare to other studies [12, 14].

Damage to aircraft from a wildlife strike in the military databases is recorded as a damage class (A/B/C/D/E/H, Table 1), based on monetary costs of repairs and human injuries rather than a categorical definition like in civil aviation. The A damage class is the most severe and H is the least [6, 8]. The USAF are required to report wildlife strikes under the Air Force Instruction 91–202 Mishap Prevention Program [9] and the Air Force Instruction 91–204 Safety Investigations Reports [21]. The USN is required to report wildlife strikes under the Commander, Navy Installations Command Instruction 3750.1 [10].

**Table 1:** Definitions of United States Navy (USN) and United States Air Force (USAF) damage classes in U.S. dollars. Damage class ‘H’ was divided for the USN dataset based on internal discussions.

Damage Class	Associated monetary Cost
A	> $2,000,000
B	$500,000 –$2,000,000
C	$50,000 –$500,000
D	$20,000 –$50,000
E	< $50,000
H (damaging)	> $55
H (non-damaging)	≤ $55

The military databases were filtered by their damage class. None of the defined damage categories are related to a strike in which no damage occurred, which is needed for the hazard ranking [6]. Therefore, strikes for which class severity was unknown in the USAF dataset were considered to be non-damaging (pers. comm. D. Sullivan, Chief, USAF BASH Team). In the USN dataset, there were no records in which strike severity was unknown. After discussions with USN personnel, we separated Class H strikes in the USN dataset into damaging (> $55 of damage costs) or non-damaging (≤ $55 of damage costs) strikes. The $55 threshold is the average cost of collecting wildlife remains from an aircraft after a strike occurs, and generally does not indicate damage to the aircraft. As these classifications differed from the civil database (minor vs. substantial damage) and were much broader, we used the median damage repair costs for civil aviation that had substantial damage to create a cutoff value between minor and substantial damage for the military dataset [6]. We then calculated separately the percentage of total strikes for each species group that resulted in damage and substantial damage [11, 14]. We were unable to use the effect-on-flight metric because these data only were recorded in the USAF and not the USN database. To increase sample size for our across-branch analysis, we combined the USN and USAF strike records, which operate similar aircraft types and are located across the U.S.

We ranked species groups as per Dolbeer et al. [6], minus the effect-on-flight metric. This procedure involved determining the percentage of total strikes for each species group that resulted in damage and substantial damage. The RHS was calculated by summing those percentages and scaling them to 100 by dividing the sum of percentages for that species by the maximum percentage for any species. Next, the species groups were ordered from most to least hazardous, including tied ranks. We then categorized airframe groups that were similar in size and flight patterns (S2 Table). Airframe groups were suggested by military aviation experts and included four groups: rotorcraft (e.g. helicopters), fighters, cargo, and stealth airframes. Some airframes could be classified in multiple categories (i.e. F-35 is considered a stealth fighter). For our purposes we grouped airframe by mission type (quick maneuverability, moving payloads, or surveillance missions), which may differ from other airframe groupings. We highlight these unique airframe groups in S2 Table. We calculated RHS for each airframe group from the combined USAF and USN dataset. We investigated differences in relative hazard scores of species groups and airframes using contingency tables and the chi-square test for goodness of fit [22].

As previous research identified avian body mass as an important predictor for RHS, we examined the relationship between RHS and avian body mass using averaged avian body mass within species [23]. For species groups, we weighted averaged body masses by the respective number of strikes for each species in the group. The bird body masses were log-transformed to normalize the data. We regressed species RHS against log-transformed avian body masses for all aircraft and the four airframe groups separately via a quadratic function or linear regression (based on best model fit). These relationships do not have biological ties, but rather relate to the physics of the mass of the bird species and the rate and pattern of influence on the RHS. We assessed model fit by the coefficient of determination (*r*^*2*^ value) and p value (α = 0.05) [24]. To emphasize the differences in airframe and flight demands that likely contribute to variance in strikes between military and civil aircraft, we descriptively compared species RHS between military and civil aircraft [12].

Lastly, we converted damage class into two binary variables for damage (1 = any level of damage occurred, 0 = no damage occurred) and for substantial damage (1 = substantial damage occurred, 0 = no substantial damage occurred) and used a binary logistic regression to evaluate how log body mass, migration flyway, reporting military branch, and airframe influenced the probability damage [14]. All variables were considered fixed effects and were included based on *a priori* hypotheses. Log body mass was a continuous variable and the remainder were categorical variables. Migration flyway included four levels: Atlantic, Central, Mississippi, and Pacific. Reporting military branch included USAF or USN. We also included interaction variables of the predictors. Candidate models were evaluated by their Akaike’s Information Criterion (AIC) with the best model having the lowest AIC [25]. We also calculated the McFadden’s *r*^2^ value as an indication of fit 1- log likelihood of the model over the log likelihood of the null model [26]. Some strikes involved more than one individual bird, and we could account for this random effect for the specific strike event by using a generalized linear mixed model approach with strike event as the random variable. However, with over 30,000 strikes, our models failed to converge. All database filtering and data analyses were conducted in R ver. 3.4.3. [27] and the ‘lme4’ and ‘arm’ packages [28,29].

## Results

From 1990–2017 an average of $20 million in damage and human injuries was attributed to wildlife strikes (*n* = 6,733) per annum for the USN in foreign and domestic operations. For the USAF in the U.S., from 1994–2017, an average of $38 million per annum in damage and human injuries was attributed to wildlife strikes (*n* = 104,129). After removing strikes that occurred in other countries, not identified to avian species, and involved a species struck more than 20 times, the combined dataset was reduced to 36,979 strikes. Of this 3,646 (10%) strike records were from the USN and 33,333 strike records (90%) came from the USAF.

The median cost for civil aviation from 2000–2015 with substantial damage was $87,570. The maximum cost associated with Class D/E/H strikes fell below $87,570, so only Class A/B/C strikes were considered as comprising substantial damage ($50,000 to ≥ $2,000,000) in our military dataset. For both military branches there were 923 strikes with substantial damage (Class A/B/C). An additional 3,024 strikes reported minor damage (Class D/E/H), which brought the total of damaging strikes to 3,947. Strikes reported with no damage (damage class unknown) represented the majority of the strike records (*n* = 33,032).

A total of 186 bird species was involved in 20 or more strikes with USN and USAF aircraft, and 3 species were struck more than 2,000 times: horned lark (*Eremophila alpestris*), mourning dove (*Zenaida macroura*), and barn swallow (*Hirundo rustica*). Birds were categorized into 108 groups. The percentage of strikes with some level of damage ranged from 1% (burrowing owl [*Athene cunicularia*]) to 74% (snow goose [*Anser caerulescens*]). There were 40 species or species groups involved in strikes with no substantial damage (Table 2). The species group involved in the highest percentage of strikes with substantial damage was the snow goose (42%). The top three species by their composite rank were snow goose, common loon (*Gavia immer*), and a tie between Canada goose (*Branta canadensis*) and black vulture (*Coragyps atratus*) (Table 2).

**Table 2:** Relative hazard scores (RHS) for 108 species groups from most to least hazardous for military aircraft within the United States.

Species	% with damage	Damage rank	% with substantial damage	Substantial damage rank	Relative hazard score (RHS)	Composite rank
Snow goose (*Anser caerulescens*)	74	1	42	1	100	1
Common loon (*Gavia immer*)	70	2	30	2	86	2
Black vulture (*Coragyps atratus*)	58	3	25	4	72	3
Canada goose (*Branta canadensis*)	56	4	29	3	74	3
Turkey vulture (*Cathartes aura*)	48	5	21	5	60	5
Northern pintail (*Anas acuta*)	44	8	16	7	51	6
Mallard (*Anas platyrhynchos*)	45	6	13	10	50	7
Swainson’s hawk (*Buteo swainsoni*)	41	9	14	8	47	8
Double-crested cormorant (*Phalacrocorax auritus*)	44	7	13	11	48	9
Herring gull (*Larus argentatus*)	32	14	18	6	43	10
Red-tailed hawk (*Buteo jamaicensis*)	37	11	14	9	43	10
Bald eagle (*Haliaeetus leucocephalus*)	40	10	11	13	44	12
*Other ducks	36	12	10	15	39	13
Pied-billed grebe (*Podilymbus podiceps*)	29	16	12	12	35	14
Great blue heron (*Ardea herodias*)	31	15	11	14	36	15
Osprey (*Pandion haliaetus*)	35	13	9	16	37	15
* Other egrets	29	18	7	19	31	17
* Other hawks	27	19	7	21	29	18
American coot (*Fulica americana*)	29	17	5	25	29	19
Ring-billed gull (*Larus delawarensis*)	19	25	8	18	23	20
Sprague’s pipit (*Anthus spragueii*)	17	28	8	17	22	21
Cattle egret (*Bubulcus ibis*)	18	26	6	22	21	22
White-winged dove (*Zenaida asiatica*)	20	23	5	26	21	23
Common grackle (*Quiscalus quiscula*)	18	27	6	24	21	24
Great horned owl (*Bubo virginianus*)	20	22	4	29	21	24
* Other falcons	15	33	7	20	19	26
Mississippi kite (*Ictinia mississippiensis*)	23	20	3	35	22	27
* Other gulls	16	30	4	28	18	28
American crow (*Corvus brachyrhynchos*)	13	37	6	23	16	29
Rock dove (*Columba livia*)	15	31	4	30	17	30
White-throated swift (*Aeronautes saxatalis*)	14	34	4	32	15	31
European starling (*Sturnus vulgaris*)	13	37	3	33	14	32
Yellow-billed Cuckoo (Coccyzus americanus)	15	32	2	38	15	32
Vaux’s swift (*Chaetura vauxi*)	12	40	4	31	14	34
Barn owl (*Tyto alba*)	12	41	3	34	12	35
Baltimore oriole (*Icterus galbula*)	10	48	2	36	11	36
* Other blackbirds	9	58	5	27	12	37
Black-bellied plover (*Pluvialis squatarola*)	13	36	1	50	12	38
Scarlet tanager (*Piranga olivacea*)	14	35	1	52	13	39
* Other doves	11	44	2	44	11	40
Purple martin (*Progne subis*)	11	42	1	48	11	41
American robin (*Turdus migratorius*)	10	49	2	43	10	42
* Other plovers	11	46	2	46	11	42
Yellow-bellied sapsucker (*Sphyrapicus varius*)	9	60	2	37	10	44
Mourning dove (*Zenaida macroura*)	9	55	2	45	9	45
Brown-headed cowbird (*Molothrus ater*)	10	47	1	55	10	46
Northern flicker (*Colaptes auratus*)	20	21	0	82	18	47
Ovenbird (*Seiurus aurocapillus*)	8	63	2	40	9	47
* Other shorebirds	9	57	1	47	9	49
* Other thrushes	11	43	1	62	10	50
Brown thrasher (*Toxostoma rufum*)	20	24	0	82	17	51
Cave swallow (*Petrochelidon fulva*)	11	45	1	61	10	51
Cardinals, grosbeaks, and allies	10	51	1	60	10	53
Red-winged blackbird (*Agelaius phoeniceus*)	7	70	2	41	8	53
Wood thrush (*Hylocichla mustelina*)	17	29	0	82	14	53
Great crested flycatcher (*Myiarchus crinitus*)	8	65	1	48	8	56
* Meadowlarks	7	71	2	42	7	56
American kestrel (*Falco sparverius*)	7	68	1	51	7	58
Gray catbird (*Dumetella carolinensis*)	10	50	0	70	9	59
Ruby-crowned kinglet (*Regulus calendula*)	7	67	1	53	7	59
Violet-green swallow (*Tachycineta thalassina*)	12	39	0	82	10	61
Scissor-tailed flycatcher (*Tyrannus forficatus*)	9	56	1	68	8	62
Sora (*Porzana carolina*)	10	52	0	82	8	63
Blue-gray gnatcatcher (*Polioptila caerulea*)	7	69	1	66	7	64
Semipalmated sandpiper (*Calidris pusilla*)	10	53	0	82	8	64
Northern mockingbird (*Mimus polyglottos*)	9	54	0	82	8	66
Killdeer (*Charadrius vociferous*)	6	81	1	59	6	67
Orchard oriole (*Icterus spurius*)	9	58	0	82	8	67
* Other flycatchers	8	61	0	82	7	69
* Other terns	4	104	2	39	5	69
* Other wood warblers	6	75	0	69	6	71
Short-eared owl (*Asio flammeus*)	8	62	0	82	7	71
Red-eyed vireo (*Vireo olivaceus*)	6	74	0	71	6	73
Barn swallow (*Hirundo rustica*)	6	79	1	67	6	74
Upland sandpiper (*Bartramia longicauda*)	8	64	0	82	7	74
House wren (*Troglodytes aedon*)	5	92	1	55	5	76
Lapland longspur (*Calcarius lapponicus*)	6	82	1	65	5	76
Dunlin (*Calidris alpine*)	8	66	0	82	7	78
Hermit thrush (*Catharus guttatus*)	6	76	0	75	6	79
Indigo bunting (*Passerina cyanea*)	5	88	1	63	5	79
Horned lark (*Eremophila alpestris*)	5	95	1	58	5	81
American goldfinch (*Spinus tristis*)	6	72	0	82	6	82
Chimney swift (*Chaetura pelagica*)	6	77	0	78	6	83
Ruby-throated hummingbird (*Archilochus colubris*)	6	73	0	82	6	83
American pipit (*Anthus rubescens*)	5	100	1	57	5	85
Cliff swallow (*Petrochelidon pyrrhonota*)	6	78	0	79	5	85
Western kingbird (*Tyrannus verticalis*)	4	105	1	54	4	87
Common nighthawk (*Chordeiles minor*)	5	96	1	64	5	88
Cedar waxwing (*Bombycilla cedrorum*)	5	84	0	77	5	89
* Other wrens	6	80	0	82	5	90
Common snipe (*Gallinago gallinago*)	6	83	0	82	5	91
* Other sparrows	5	94	0	72	5	92
Golden-crowned kinglet (*Regulus satrapa*)	5	85	0	82	5	93
Bank swallow (*Riparia riparia*)	5	86	0	82	4	94
* Other vireos	5	87	0	82	4	95
Lesser nighthawk (*Chordeiles acutipennis)*	5	89	0	82	4	96
Least sandpiper (*Calidris minutilla*)	5	90	0	82	4	97
Yellow-rumped warbler (*Dendroica coronate*)	5	91	0	81	4	97
Common yellowthroat (*Geothlypis trichas*)	5	99	0	74	4	99
House finch (*Haemorhous mexicanus*)	5	92	0	82	4	100
Snow bunting (*Plectrophenax nivalis*)	5	97	0	82	4	101
* Other longspurs	5	98	0	82	4	102
Savannah sparrow (*Passerculus sandwichensis*)	4	107	0	73	4	102
Dark-eyed junco (*Junco hyemalis*)	4	106	0	75	4	104
Tree swallow (*Tachycineta bicolor*)	5	101	0	80	4	104
Bobolink (*Dolichonyx oryzivorus*)	4	102	0	82	4	106
Wilson’s Snipe (*Gallinago delicate*)	4	103	0	82	4	107
Burrowing owl (*Athene cunicularia*)	1	108	0	82	1	108

The composite rank represents the sum of the percentage of strikes with damage and the percentage of strikes with substantial damage for that species group against all species.

* denotes a species group.

See S1 Table for a list of species in each species group (i.e. Other ducks). Strike data are from separate databases maintained by the USN (1990–2017) and USAF (1994–2017).

**Fig 1:**
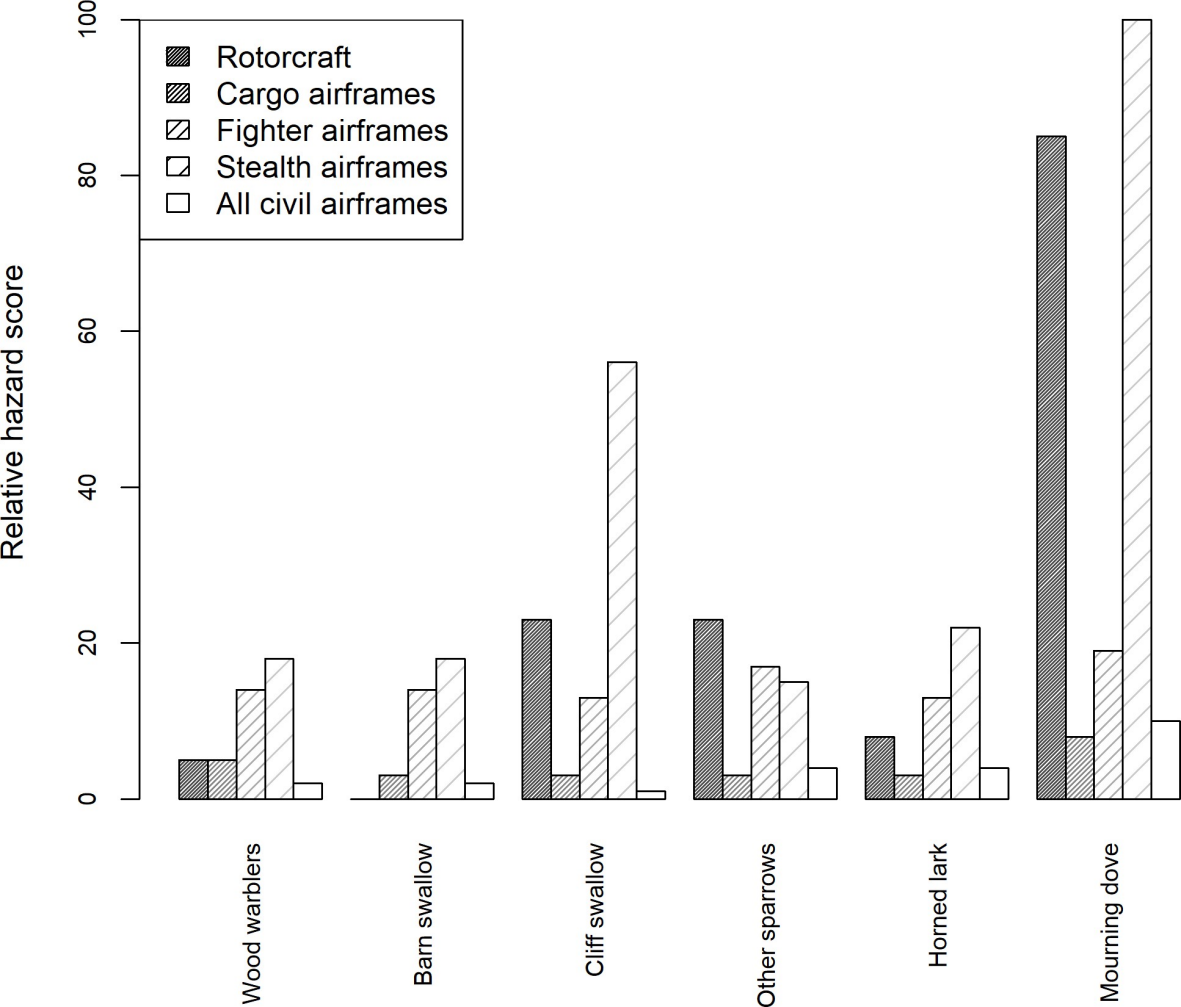
Comparison of relative hazard scores for 6 avian species groups for 4 military airframe groups and all civil airframes for 6 species. Relative hazard scores are calculated from bird strikes within the U.S. See S2 Table for airframe group compositions. Civil relative hazard scores come from [11]. Species are ordered from left to right by ascending averaged body mass.

Filtering the strike records to only fighter, cargo, stealth, and rotorcraft and to species or species groups that were struck over 20 times reduced the total number of strike records to 31,082. There were 9,535 strike records for military fighter airframes (65 species struck over 20 times, S3 Table), 20,174 records for cargo airframes (98 species struck over 20 times, S4 Table), 674 strike records for stealth airframes (12 species struck over 20 times, S5 Table), and 699 strike records for rotorcraft (16 species struck over 20 times, S6 Table). There were 64 species groups that were similar for fighter and cargo airframes; however, only 6 species were represented across all 4 aircraft groups and the civil dataset. RHS for the 6 species and species groups across airframe groups differed significantly (χ^2^_*20*_ = 105.11 *P* < 0.001, Fig 1). Of the 6 species groups compared among airframe groups, all had higher RHS for the stealth than all civil airframes.

**Fig 2:**
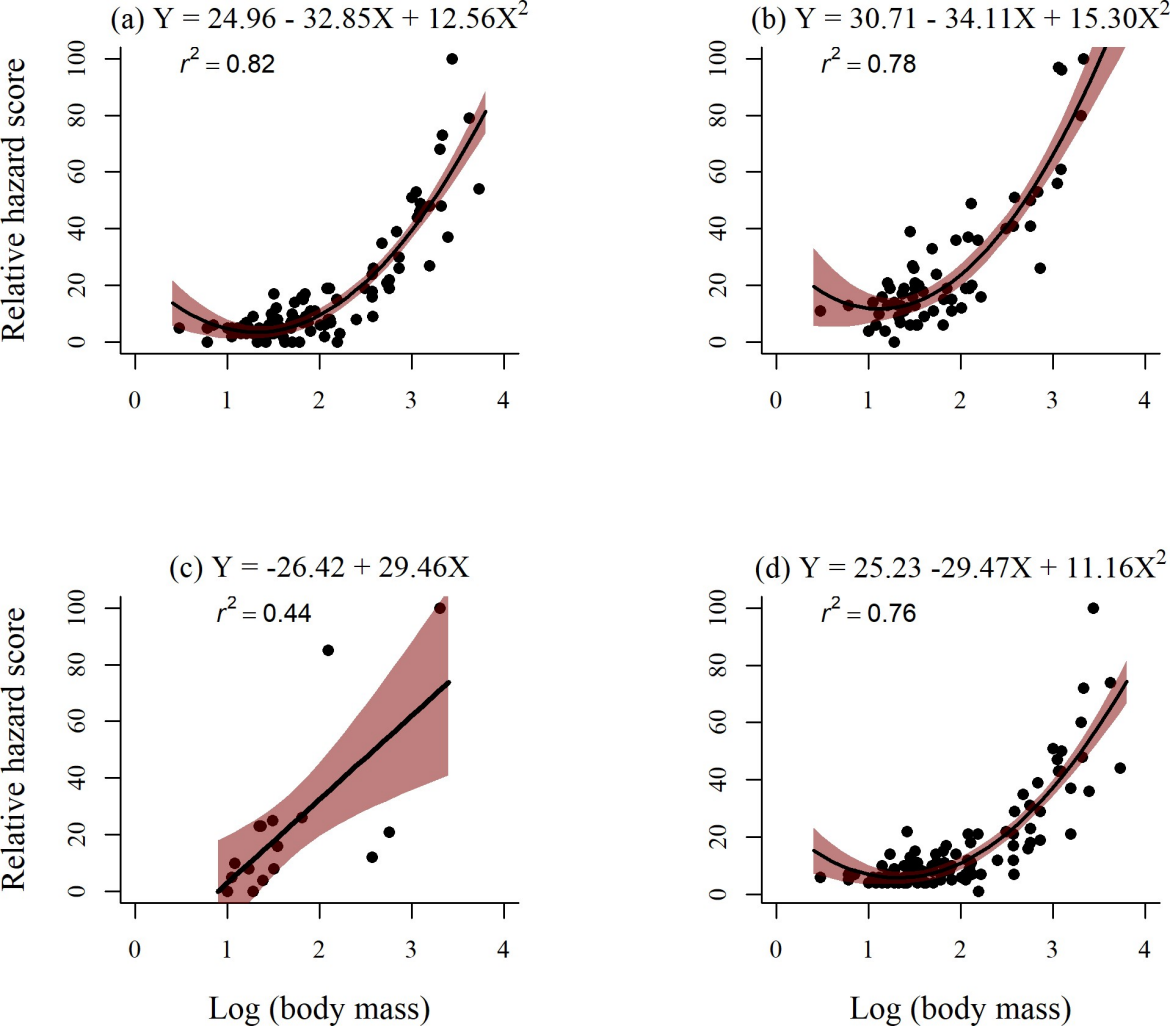
**Relationship between avian body mass and relative hazard score for avian groups with military cargo (a), fighter (b), rotorcraft (c) and all military airframes (d).** Only strikes identified to species and occurred within the U.S. were included. Strike data are from separate databases maintained by the U.S. Navy (1990–2017) and U.S. Air Force (1994–2017). Equations and coefficient of determination (*r*^*2*^ values) and 95% confidence intervals are displayed.

There was a strong positive quadratic relationship between RHS and avian body mass for the species groups involved in bird strikes with all military aircraft (*r*^*2*^ = 0.76, Fig 2(D). The quadratic curve was steeper for the fighter airframe with a higher y-intercept, compared to the cargo trend (Fig 2). The best fit for rotorcraft and log avian body mass was a linear regression (Fig 2(C), whereas the quadratic relationship was the best fit for fighter and cargo airframes (Fig 2(A) and 2(B), S7 Table). Stealth airframes did not exhibit a relationship with avian body mass regardless of equations used and is not included in Fig 2.

**Fig 3:**
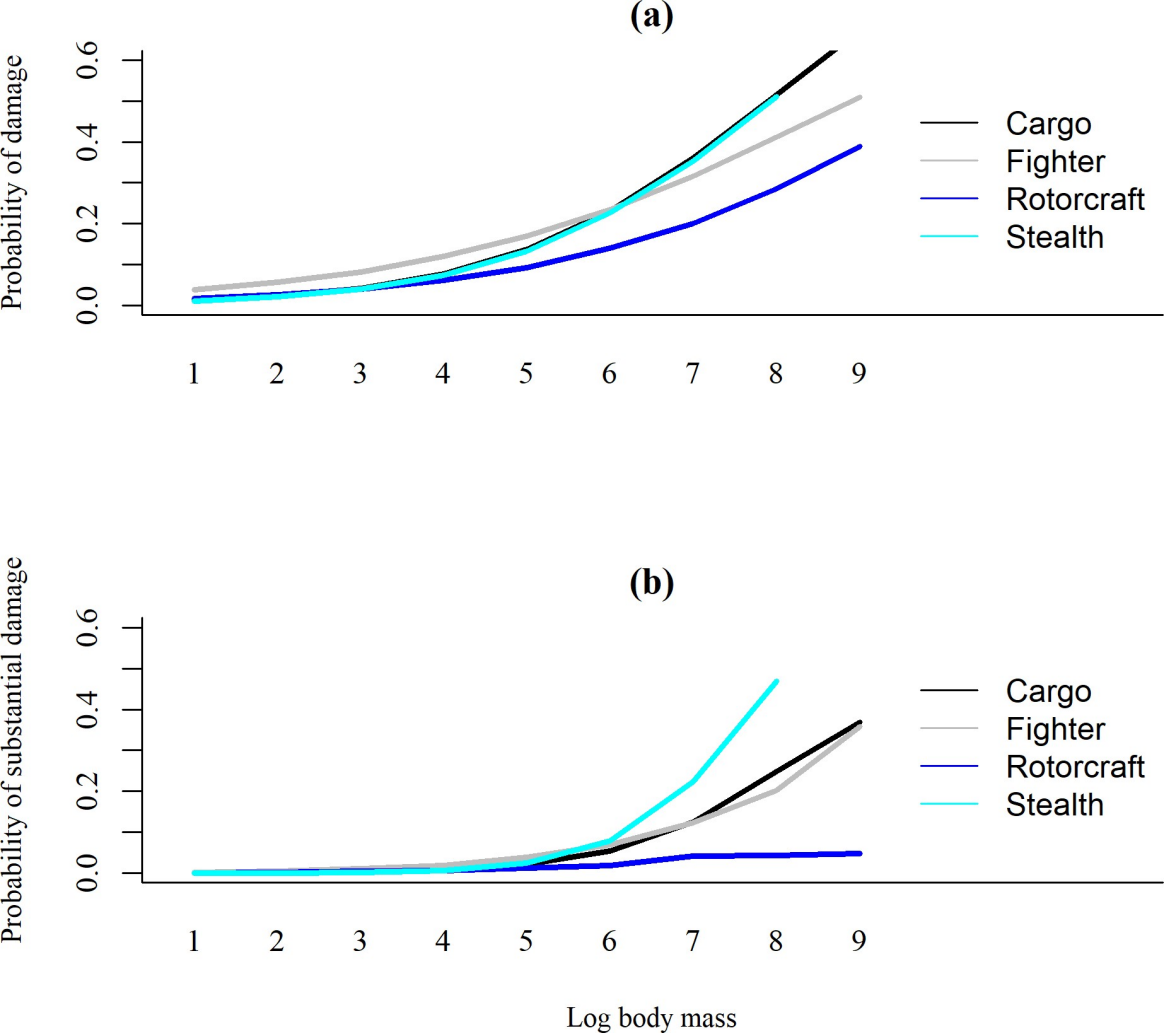
**Interaction plot of airframes, avian log body mass, and predicted probability of damage (a) and substantial damage (b).** Only strikes identified to species and occurred within the U.S. were included. Strike data are from separate databases maintained by the United States Navy (1990–2017) and Air Force (1994–2017).

The best logistic regression models predicting the probability of damage was the same for any damage and substantial damage and included all predictor variables and the airframe × avian body mass interaction (S8 Table). Probability of any damage and substantial damage increased with avian body mass. Within airframe type, fighters had the highest probability of damage or substantial damage compared to cargo airframes (Tables 3 & 4). Probability of damage or substantial damage was influenced by avian body mass, and this effect differed per airframe. These probabilities differed per airframe type for bird species with the same body mass (Fig 3). Migration flyway, specifically the Mississippi flyway, had the lowest probability of substantial damage compared to the Atlantic flyway (Table 4). Reporting military branch also contributed to the probability of substantial damage. USAF aircraft were predicted to have a greater probability of a strike with substantial damage than USN aircraft (Table 4).

**Table 3:** Binary logistic regression models predicting any level of damage to Navy (USN) and Air Force (USAF) within the United States. This model represents the best as evaluated by the lowest Akaike’s Information Criterion (AIC) value (see S8 Table for other candidate models). McFadden’s *r*^2^ value is 0.12. Predictor variables include migration flyway (Central, Mississippi, Pacific, Atlantic), airframe (cargo, rotorcraft, stealth, fighter), avian log body mass, military branch (USN or USAF), and the airframe × avian log body mass interaction.

			Confidence intervals	
Parameter*	Coefficient	SE	2.5%	97.5%
Intercept	-5.28	0.09	-5.45	-5.11
Central	0.05	0.05	-0.05	0.14
Mississippi	-0.06	0.05	-0.17	0.04
Pacific	-0.09	0.06	-0.21	0.03
Fighter	1.45	0.13	1.20	1.70
Rotorcraft	0.59	0.36	-0.14	1.27
Stealth	-0.01	0.39	-0.79	0.74
Log mass	0.70	0.02	0.66	0.73
USN	0.04	0.07	-0.10	0.18
Fighter × log mass	-0.24	0.02	-0.29	-0.20
Rotorcraft × log mass	-0.22	0.07	-0.35	-0.08
Stealth × log mass	0.00	0.08	-0.16	0.14

*Reference categories include Atlantic flyway, cargo airframe, USAF branch, and the interaction term cargo × log mass.

**Table 4:** Binary logistic regression models predicting substantial damage to Navy (USN) and Air Force (USAF) within the United States. This model represents the best as evaluated by the lowest Akaike’s Information Criterion (AIC) value (see S8 Table for other candidate models). McFadden’s *r*^2^ value is 0.23. Predictor variables include flyway (Central, Mississippi, Pacific, Atlantic), airframe (cargo, rotorcraft, stealth, fighter), avian log body mass, military branch, and the airframe × avian log body mass interaction.

			Confidence intervals	
Parameter*	Coefficient	SE	2.5%	97.5%
Intercept	-8.54	0.21	-8.96	-8.13
Central	-0.18	0.09	-0.37	0.01
Mississippi	-0.31	0.10	-0.51	-0.10
Pacific	0.19	0.11	-0.02	0.40
Fighter	1.94	0.29	1.37	2.50
Rotorcraft	1.42	1.02	-0.83	3.26
Stealth	-1.47	1.05	-3.77	0.39
Log mass	0.98	0.03	0.91	1.04
USN	-1.73	0.25	-2.25	-1.28
Fighter × log mass	-0.26	0.05	-0.35	-0.17
Rotorcraft × log mass	-0.32	0.18	-0.67	0.06
Stealth × log mass	0.32	0.16	0.03	0.66

*Reference categories include Atlantic flyway, cargo airframe, USAF branch, and the interaction term cargo× log mass.

## Discussion

Ranking of wildlife hazards is an essential component of strike risk [12]. Although we did not estimate strike risk in this study, these RHS can help guide future research in developing airframe-specific risk metrics for military airfields. We predicted that airframe and avian body mass, migration flyway, and reporting military branch would influence the relative hazard scores and the probability of damage.

For the combined USAF and USN datasets, there was a strong quadratic trend between avian body mass and RHS. As avian body mass increased, so too did RHS, as observed in similar studies [11]. This significant trend can be used to calculate RHS for species not included in our dataset (i.e. equations in Fig 2 can be solved for RHS with the given body mass of the bird species). We caution the use of the linear equation identified for rotorcraft for predicting RHS because of the low coefficient of determination (*r*^*2*^ value). The relationship between airframes and RHS, however, would be useful for the few species that are not included in the dataset because of the high coefficient of determination.

The stealth airframes had higher relative hazard scores compared to civil airframe groups. Interestingly, for 6 species struck by both military and civil aircraft, all had higher RHS for stealth than civil airframes. The mourning dove had a military RHS of 100 for stealth, but a score of 9 for civil aircraft [11]. We suspect that the materials composing stealth aircraft “skin” and specialized aircraft components have elevated sensitivity to strikes. For example, a strike in 2006 which involved a mourning dove (123 g) resulted in $129,787 damage to the wing of a stealth bomber. Mourning doves are capable of causing similar amounts of damage (> $130,000) for civil aircraft [7]; however, all of the reported mourning dove strikes from 2010–2015 involved the bird being ingesting into an engine, not external wing damage.

Relative hazard scores for fighter airframes were generally higher than cargo and all civil airframes. Furthermore, our logistic regression models indicated that fighter airframes experienced a higher probability of damage with birds of a lower body mass compared to cargo airframes. It is likely that higher speeds and lower flight altitudes of fighter airframes versus cargo aircraft contributed to higher RHS and probability of damage. Cargo airframes are likely similar to civil aviation in terms of design and flight patterns, with long ascents and descents and high cruising altitudes [8, 15]. All airframes, except rotorcraft, exhibited an exponential relationship between avian body mass and RHS. This relationship indicates that below a certain body mass, the RHS exhibits the opposite trend of increasing with decreasing body mass. We suggest that this trend is not biologically relevant, but a byproduct of the mathematical analysis such as from the numerous outliers. Furthermore, there are few RHS for birds with extremely small body masses. Interestingly, the trend between body mass and RHS for rotorcraft was not a strong relationship and the best fit was linear. Possible explanations for this result include differences in strike reporting rates, flight patterns, or airframe components. For example, strikes to wind screens, rotors, and tail rotors, components that are quite exposed, can result in frequent, substantial damage [30]

We acknowledge that these RHS are based on a degree of frequency (species only included if struck 20 or more times) and thus influenced by differences in strike rates across species. Certain species are struck more frequently than others. Frequently struck birds use the airport for foraging and roosting activities [3] and generally are more maneuverable [31]. However, there is significant individual and species variability [32, 33] that influence avian responses to oncoming aircraft. Given this caveat, our results were similar to other studies in that birds associated with water were ranked as most hazardous [6, 8, 11, 14], as well as raptors [6, 8, 11].

Our results indicate that avian hazards to military aviation are airframe specific. Depending on the airfield, hazard scores can be calculated per airframe type. For example, if an airfield is primarily used for fighter missions/training, the fighter airframe RHS should be used, ensuring that species on the airfield would be prioritized based on the severity of the strike specific to that airframe. These results, coupled with data on species seasonal relative abundance on and near airfields [30, 34], will be important in directing airfield management [12]. Ultimately, the development of airframe-specific strike risk metrics will yield more accurate information on airframe vulnerability to bird strikes by mission type.

## Conclusion

We found that avian hazards to military aviation differed from civil aviation. Specifically, species RHS were higher for military than civil aircraft. Furthermore, we found differences in RHS among airframe groups. As predicted, airframes that travel at greater speeds (fighters) experienced greater damage from birds when struck, especially at the lower end of the body mass scale. Although these results are based on strikes within and outside of the airfield environment, the calculated hazard scores can be used in conjunction with a frequency component to estimate risk.

## Supporting information

S1 Table Bird species (*n* = 186) involved in more than 20 strikes with military aircraft grouped into 108 species groups. Species with fewer than 50 strikes were combined into species groups based on phylogeny.
(DOCX)

S2 Table Airframe groups (*n* = 189) involved in bird strikes with military aircraft grouped into 8 airframe groups. (DOCX)

S3 Table Relative hazard scores (RHS) for 65 avian species groups from most to least hazardous for fighter aircraft within the United States. (DOCX)

S4 Table Relative hazard scores (RHS) for 65 species groups from most to least hazardous for cargo aircraft within the United States. (DOCX)

S5 Table Relative hazard scores (RHS) for 12 species groups from most to least hazardous for stealth aircraft within the United States. (DOCX)

S6 Table Relative hazard scores (RHS) for 16 species groups from most to least hazardous for stealth aircraft within the United States. (DOCX)

S7 Table Summary of quadratic and linear relationships with military airframe and avian log body mass. No significant relationship was found for stealth airframes and avian log body mass. Bold values indicate best fit by the coefficient of determination (*r*^*2*^ values).
(DOCX)

S8 Table Results from the binomial generalized linear model of factors that influence the probability of a bird strike to cause (a) any damage and the probability of a bird strike causing (b) substantial damage with military aircraft.
(DOCX)
