# Supplementary material for: Quantification of avian hazards to military aircraft and implications for wildlife management
Source: PLoS One. 2018 Nov 1;13(11):e0206599. doi: 10.1371/journal.pone.0206599 (PMC6211720; doi:10.1371/journal.pone.0206599)
Supplement: S1 Table — Species with fewer than 50 strikes were combined into species groups based on phylogeny. (DOCX) [file pone.0206599.s001.docx]

**S1 Table.** **Bird species (*n* =186) involved in more than 20 strikes with military aircraft grouped into 108 species groups. Species with fewer than 50 strikes were combined into species groups based on phylogeny.**

| Acadian flycatcher | Other flycatchers |
| --- | --- |
| American coot | American coot |
| *American crow | American crow |
| American golden-plover | Other plovers |
| American goldfinch | American goldfinch |
| American kestrel | American kestrel |
| American pipit | American pipit |
| American redstart | Other wood warblers |
| American robin | American robin |
| American tree sparrow | Other sparrows |
| American wigeon | Other ducks |
| American woodcock | Other shorebirds |
| Baird’s sandpiper | Other shorebirds |
| *Bald eagle | Bald eagle |
| Baltimore oriole | Baltimore oriole |
| Bank swallow | Bank swallow |
| Barn owl | Barn owl |
| Barn swallow | Barn swallow |
| Bay-breasted warbler | Other wood warblers |
| Black tern | Other terns |
| Black vulture | Black vulture |
| Black-and-white warbler | Other wood warblers |
| Black-bellied plover | Black-bellied plover |
| Blackburnian warbler | Other wood warblers |
| Blackpoll warbler | Other wood warblers |
| Black-throated blue warbler | Other wood warblers |
| Black-throated green warbler | Other wood warblers |
| Black-throated sparrow | Other sparrows |
| Blue-gray gnatcatcher | Blue-gray gnatcatcher |
| Blue-headed vireo | Other vireos |
| Blue-winged teal | Other ducks |
| Bobolink | Bobolink |
| Brewer’s blackbird | Other blackbirds |
| Brewer’s sparrow | Other sparrows |
| Broad-winged hawk | Other hawks |
| Brown thrasher | Brown thrasher |
| Brown-headed cowbird | Brown-headed cowbird |
| Buff-breasted sandpiper | Other shorebirds |
| Burrowing owl | Burrowing owl |
| California gull | Other gulls |
| Canada goose | Canada goose |
| Canada warbler | Other wood warblers |
| Cape may warbler | Other wood warblers |
| Cassin’s sparrow | Other sparrows |
| Cattle egret | Cattle egret |
| Cave swallow | Cave swallow |
| Cedar waxwing | Cedar waxwing |
| Chestnut-collared longspur | Other longspurs |
| Chestnut-sided warbler | Other wood warblers |
| Chimney swift | Chimney swift |
| Chipping sparrow | Other sparrows |
| Clay-colored sparrow | Other sparrows |
| Cliff swallow | Cliff swallow |
| Common grackle | Common grackle |
| Common ground dove | Other doves |
| *Common loon | Common loon |
| Common nighthawk | Common nighthawk |
| Common snipe | Common snipe |
| Common yellowthroat | Common yellowthroat |
| Coopers hawk | Other hawks |
| Dark-eyed junco | Dark-eyed junco |
| Dickcissel | Cardinals, grosbeaks, and allies |
| *Double-crested cormorant | Double-crested cormorant |
| Dunlin | Dunlin |
| Eastern bluebird | Other thrushes |
| Eastern kingbird | Other flycatchers |
| Eastern meadowlark | Meadowlarks |
| Eastern towhee | Other sparrows |
| Eastern wood-pewee | Other flycatchers |
| Eurasian collared dove | Other doves |
| European starling | European starling |
| Ferruginous hawk | Other hawks |
| Field sparrow | Other sparrows |
| Fox sparrow | Other sparrows |
| Franklin’s gull | Other gulls |
| Gadwall | Other ducks |
| *Golden-crowned kinglet | Golden-crowned kinglet |
| Grasshopper sparrow | Other sparrows |
| Gray catbird | Gray catbird |
| Gray-cheeked thrush | Other thrushes |
| Great blue heron | Great blue heron |
| Great crested flycatcher | Great crested flycatcher |
| Great egret | Other egrets |
| Great horned owl | Great horned owl |
| Great-tailed grackle | Other blackbirds |
| Green-winged teal | Other ducks |
| Green heron | Green heron |
| Hermit thrush | Hermit thrush |
| Herring gull | Herring gull |
| Hooded warbler | Other wood warblers |
| Horned lark | Horned lark |
| House finch | House finch |
| House sparrow | Other sparrows |
| House wren | House wren |
| Indigo bunting | Indigo bunting |
| Killdeer | Killdeer |
| Lapland longspur | Lapland longspur |
| Lark bunting | Other sparrows |
| Lark sparrow | Other sparrows |
| Laughing gull | Other gulls |
| Least flycatcher | Other flycatchers |
| Least sandpiper | Least sandpiper |
| Least tern | Other terns |
| Leconte’s sparrow | Other sparrows |
| Lesser nighthawk | Lesser nighthawk |
| Lesser scaup | Other ducks |
| Lincoln’s sparrow | Other sparrows |
| Magnolia warbler | Other wood warblers |
| Mallard | Mallard |
| Marsh wren | Other wrens |
| Mccown’s longspur | Other longspurs |
| Merlin | Other falcons |
| Mississippi kite | Mississippi kite |
| Mourning dove | Mourning dove |
| Nashville warbler | Other wood warblers |
| *Northern flicker | Northern flicker |
| Northern harrier | Other hawks |
| Northern mockingbird | Northern mockingbird |
| Northern parula | Other wood warblers |
| Northern pintail | Northern pintail |
| Northern shoveler | Other ducks |
| Northern waterthrush | Other wood warblers |
| Orange-crowned warbler | Other wood warblers |
| *Orchard oriole | Orchard oriole |
| Osprey | Osprey |
| Ovenbird | Ovenbird |
| Pacific golden plover | Other plovers |
| Pacific-slope flycatcher | Other flycatchers |
| Palm warbler | Other wood warblers |
| Pectoral sandpiper | Other shorebirds |
| Peregrine falcon | Other falcons |
| *Pied-billed grebe | Pied-billed grebe |
| Pine warbler | Other wood warblers |
| Prairie warbler | Other wood warblers |
| Purple martin | Purple martin |
| Red-eyed vireo | Red-eyed vireo |
| Red-shouldered hawk | Other hawks |
| Red-tailed hawk | Red-tailed hawk |
| Red-winged blackbird | Red-winged blackbird |
| Ring-billed gull | Ring-billed gull |
| Rock dove | Rock dove |
| Rose-breasted grosbeak | Cardinals, grosbeaks, and allies |
| Ruby-crowned kinglet | Ruby-crowned kinglet |
| Ruby-throated hummingbird | Ruby-throated hummingbird |
| Ruddy duck | Other ducks |
| Sage sparrow | Other sparrows |
| Savannah sparrow | Savannah sparrow |
| Scarlet tanager | Scarlet tanager |
| Scissor-tailed flycatcher | Scissor-tailed flycatcher |
| Semipalmated plover | Other plovers |
| *Semipalmated sandpiper | Semipalmated sandpiper |
| Sharp-shinned hawk | Other hawks |
| Short-eared owl | Short-eared owl |
| Smith’s longspur | Other longspurs |
| Snow bunting | Snow bunting |
| Snow goose | Snow goose |
| Song sparrow | Other sparrows |
| Sora | Sora |
| *Sprague’s pipit | Spragues pipit |
| Swainson’s hawk | Swainsons hawk |
| Swainson’s thrush | Other thrushes |
| Swamp sparrow | Other sparrows |
| Tennessee warbler | Other wood warblers |
| Townsends warbler | Other wood warblers |
| Tree swallow | Tree swallow |
| Turkey vulture | Turkey vulture |
| Upland sandpiper | Upland sandpiper |
| *Vaux’s swift | Vauxs swift |
| Veery | Other thrushes |
| Vesper sparrow | Other sparrows |
| *Violet-green swallow | Violet-green swallow |
| Warbling vireo | Other vireos |
| Western kingbird | Western kingbird |
| Western meadowlark | Meadowlarks |
| Western sandpiper | Other shorebirds |
| Western tanager | Cardinals, grosbeaks, and allies |
| White-crowned sparrow | Other sparrows |
| White-eyed vireo | Other vireos |
| White-throated sparrow | Other sparrows |
| White-throated swift | White-throated swift |
| White-winged dove | White-winged dove |
| Wilson’s snipe | Wilson's snipe |
| Wilson’s warbler | Other wood warblers |
| Winter wren | Other wrens |
| Wood duck | Other ducks |

* denotes a species with fewer than 50 strikes that could not be grouped with related species.
