# Supplementary material for: Quantification of avian hazards to military aircraft and implications for wildlife management
Source: PLoS One. 2018 Nov 1;13(11):e0206599. doi: 10.1371/journal.pone.0206599 (PMC6211720; doi:10.1371/journal.pone.0206599)
Supplement: S2 Table — (DOCX) [file pone.0206599.s002.docx]

**S2 Table.** **Airframe groups (*n* = 189) involved in bird strikes with military aircraft grouped into 8 airframe groups.**

| Airframe |  | Airframe group |
| --- | --- | --- |
| C-130 |  | heavy |
| C-135 |  | heavy |
| C-17 |  | heavy |
| F-16 |  | fighter |
| T-38 |  | fighter |
| T-1 |  | fighter |
| A-10 |  | heavy |
| F-15 |  | fighter |
| C-5 |  | heavy |
| KC-10 |  | heavy |
| T-6 |  | fighter |
| T045C |  | fighter |
| B-52 |  | heavy |
| B-2 |  | stealth |
| T-37 |  | fighter |
| C-21 |  | heavy |
| E-8 |  | heavy |
| B-1* |  | stealth |
| H-60 |  | rotorcraft |
| F-22 |  | fighter |
| TC012B |  | fighter |
| F018F |  | fighter |
| H-1 |  | rotorcraft |
| P008A |  | heavy |
| P003C |  | heavy |
| T006B |  | propeller/small |
| E-3 |  | heavy |
| F018E |  | fighter |
| E-4 |  | heavy |
| C-141 |  | heavy |
| T034C |  | propeller/small |
| F018C |  | fighter |
| C-40 |  | heavy |
| V-22 |  | rotorcraft |
| C-9 |  | heavy |
| C-32 |  | heavy |
| TH057C |  | rotorcraft |
| MH060S |  | rotorcraft |
| C-12 |  | propeller/small |
| MH060R |  | rotorcraft |
| T045A |  | rotorcraft |
| T044C |  | propeller/small |
| E006B |  | heavy |
| T044A |  | propeller/small |
| C-37 |  | propeller/small |
| MV022B |  | rotorcraft |
| F-35* |  | fighter |
| EA006B |  | heavy |
| T006A |  | propeller/small |
| KC130J |  | heavy |
| U-2* |  | stealth |
| C-20 |  | heavy |
| U-28 |  | propeller/small |
| H-53 |  | rotorcraft |
| C-146 |  | propeller/heavy |
| EA018G |  | fighter |
| SH060B |  | rotorcraft |
| T-43 |  | heavy |
| E002C+ |  | propeller/heavy |
| T039N |  | fighter |
| F005N |  | fighter |
| T-53 |  | rotorcraft |
| F018D |  | fighter |
| F-117 |  | stealth |
| C026 |  | propeller/small |
| CH053E |  | rotorcraft |
| C040A |  | heavy |
| E002C |  | rotorcraft |
| AV008B |  | fighter |
| C130T |  | propeller/heavy |
| C-26 |  | propeller/small |
| E002D |  | rotorcraft |
| F-4 |  | fighter |
| C002A |  | propeller/heavy |
| C-145 |  | propeller/small |
| C009B |  | heavy |
| F018 |  | fighter |
| AH001W |  | rotorcraft |
| F018A+ |  | fighter |
| T038C |  | fighter |
| T044 |  | propeller/small |
| F035B |  | stealth |
| UH001Y |  | rotorcraft |
| F-18 |  | fighter |
| Q-9 |  | propeller/heavy |
| F018B |  | fighter |
| SH060F |  | rotorcraft |
| C130A |  | propeller/heavy |
| CRJ |  | heavy |
| F015C |  | fighter |
| F035C |  | fighter |
| OTHER |  | Other |
| C-27 |  | propeller/heavy |
| Q-4 |  | stealth |
| NP003D |  | propeller/heavy |
| TH057B |  | rotorcraft |
| UC012B |  | propeller/heavy |
| E-9 |  | propeller/heavy |
| UV-18 |  | propeller/heavy |
| T-41 |  | propeller/heavy |
| C130F |  | propeller/heavy |
| F018A |  | fighter |
| KC130F |  | propeller/heavy |
| MH053E |  | rotorcraft |
| F-111 |  | fighter |
| CC |  | heavy |
| CH046E |  | rotorcraft |
| F005F |  | fighter |
| TAV008B |  | fighter |
| AL-1 |  | heavy |
| C-38 |  | heavy |
| E-6 |  | heavy |
| C130 |  | propeller/heavy |
| F016A |  | fighter |
| F016C |  | fighter |
| T039G |  | heavy |
| C-18 |  | heavy |
| EXEC/CORP |  | Other |
| T-3 |  | propeller/small |
| TG-10 |  | propeller/heavy |
| TG-16 |  | propeller/heavy |
| AH001Z |  | rotorcraft |
| C017A |  | heavy |
| C035D |  | heavy |
| C130J |  | propeller/heavy |
| HH065 |  | rotorcraft |
| KC130T |  | propeller/heavy |
| P003 |  | propeller/heavy |
| S003B |  | fighter |
| T006 |  | propeller/small |
| T039D |  | heavy |
| UH060L |  | rotorcraft |
| C-137 |  | heavy |
| C-22 |  | heavy |
| T-51 |  | propeller/small |
| T-52 |  | propeller/small |
| AH001 |  | rotorcraft |
| BO707 |  | heavy |
| C012 |  | propeller/small |
| C020D |  | heavy |
| C026A |  | propeller/small |
| C130H |  | propeller/heavy |
| CP |  | Other |
| EP003 |  | propeller/heavy |
| F016 |  | fighter |
| F016B |  | fighter |
| F016N |  | fighter |
| HH060H |  | rotorcraft |
| UH001N |  | rotorcraft |
| UH003H |  | rotorcraft |
| C-150 |  | propeller/small |
| DA-20 |  | propeller/small |
| PA-32 |  | propeller/small |
| T-39 |  | heavy |
| TG-7 |  | propeller/small |
| A004F |  | fighter |
| AV008 |  | fighter |
| B200 |  | propeller/small |
| BA146 |  | heavy |
| BO747 |  | heavy |
| C009 |  | heavy |
| C012B |  | propeller/small |
| C012C |  | propeller/small |
| C012F |  | propeller/small |
| C017 |  | heavy |
| C020G |  | heavy |
| C040 |  | heavy |
| CH046 |  | rotorcraft |
| EC130J |  | propeller/heavy |
| F015D |  | fighter |
| F022A |  | fighter |
| F035A |  | fighter |
| HH001N |  | rotorcraft |
| HH060 |  | rotorcraft |
| HH065A |  | rotorcraft |
| KC135T |  | heavy |
| NC-12B |  | propeller/small |
| S003 |  | fighter |
| UC012F |  | propeller/small |
| UC012M |  | propeller/small |
| VH060N |  | rotorcraft |
| C-25 |  | propeller/small |
| Q-1 |  | Other |
| TG-14 |  | Other |
| OTHER |  | Other |
| Aerostat |  | Other |
| UNKNOWN |  | Other |
| Private |  | Other |
| Raven |  | Other |
