# Supplementary material for: Quantification of avian hazards to military aircraft and implications for wildlife management
Source: PLoS One. 2018 Nov 1;13(11):e0206599. doi: 10.1371/journal.pone.0206599 (PMC6211720; doi:10.1371/journal.pone.0206599)
Supplement: S3 Table — (DOCX) [file pone.0206599.s003.docx]

**S3 Table. Relative hazard scores (RHS) for 65 species groups from most to least hazardous for fighter aircraft within the United States.**

| Species | % with damage | Damage rank | % with substantial damage | Substantial damage rank | Relative hazard score  (RHS) | Composite rank |
| --- | --- | --- | --- | --- | --- | --- |
| Black vulture (*Coragyps atratus*) | 55 | 1 | 23 | 3 | 100 | 1 |
| Herring gull (*Larus argentatus*) | 44 | 3 | 32 | 1 | 97 | 1 |
| Mallard (*Anas platyrhynchos*) | 50 | 2 | 25 | 2 | 96 | 1 |
| Turkey vulture (*Cathartes aura*) | 42 | 4 | 21 | 4 | 80 | 4 |
| Red-tailed hawk (*Buteo jamaicensis*) | 34 | 6 | 13 | 5 | 61 | 5 |
| * Other ducks | 31 | 8 | 10 | 7 | 53 | 6 |
| Swainson’s hawk (*Buteo swainsoni*) | 36 | 5 | 8 | 10 | 56 | 6 |
| Ring-billed gull (*Larus delawarensis*) | 26 | 10 | 13 | 6 | 50 | 8 |
| * Other plovers | 29 | 9 | 10 | 9 | 49 | 9 |
| * Other hawks | 34 | 7 | 6 | 15 | 51 | 10 |
| * Other gulls | 24 | 14 | 8 | 11 | 41 | 11 |
| Rock dove (*Columba livia*) | 25 | 13 | 7 | 12 | 41 | 11 |
| Common grackle (*Quiscalus quiscula*) | 19 | 20 | 10 | 8 | 37 | 13 |
| Mississippi kite (*Ictinia mississippiensis*) | 26 | 12 | 6 | 16 | 40 | 13 |
| Scarlet tanager (*Piranga olivacea*) | 26 | 11 | 4 | 18 | 39 | 15 |
| European starling (*Sturnus vulgaris*) | 22 | 16 | 6 | 14 | 36 | 16 |
| White-winged dove (*Zenaida asiatica*) | 21 | 17 | 7 | 13 | 36 | 16 |
| Brown-headed cowbird (*Molothrus ater*) | 22 | 15 | 4 | 20 | 33 | 18 |
| * Other thrushes | 17 | 21 | 3 | 22 | 26 | 19 |
| Purple martin (*Progne subis*) | 16 | 23 | 3 | 23 | 24 | 20 |
| White-throated swift (*Aeronautes saxatalis*) | 13 | 31 | 4 | 21 | 21 | 21 |
| American kestrel (*Falco sparverius*) | 14 | 25 | 2 | 31 | 20 | 22 |
| * Meadowlarks | 11 | 37 | 4 | 19 | 19 | 22 |
| Red-eyed vireo (*Vireo olivaceus*) | 13 | 29 | 2 | 27 | 19 | 22 |
| Gray catbird (*Dumetella carolinensis*) | 14 | 26 | 2 | 32 | 20 | 25 |
| * Other shorebirds | 13 | 30 | 2 | 28 | 19 | 25 |
| Hermit thrush (*Catharus guttatus*) | 21 | 18 | 0 | 44 | 27 | 27 |
| * Other sparrows | 11 | 36 | 2 | 26 | 17 | 27 |
| American coot (*Fulica americana*) | 20 | 19 | 0 | 44 | 26 | 29 |
| Cave swallow (*Petrochelidon fulva*) | 14 | 27 | 1 | 37 | 19 | 30 |
| Mourning dove (*Zenaida macroura*) | 12 | 35 | 2 | 29 | 19 | 30 |
| * Other vireos | 17 | 22 | 0 | 44 | 21 | 32 |
| Scissor-tailed flycatcher (*Tyrannus forficatus*) | 13 | 28 | 1 | 39 | 18 | 33 |
| Great crested flycatcher (*Myiarchus crinitus*) | 15 | 24 | 0 | 44 | 19 | 34 |
| Common nighthawk (*Chordeiles minor*) | 9 | 45 | 3 | 24 | 15 | 35 |
| Red-winged blackbird (*Agelaius phoeniceus*) | 7 | 52 | 4 | 17 | 15 | 35 |
| Bank swallow (*Riparia riparia*) | 13 | 31 | 0 | 44 | 16 | 37 |
| Barn Swallow (*Hirundo rustica*) | 10 | 39 | 1 | 36 | 14 | 37 |
| Cardinals, grosbeaks, and allies | 13 | 31 | 0 | 44 | 16 | 37 |
| Other wood warblers | 10 | 41 | 1 | 34 | 14 | 37 |
| Upland sandpiper (*Bartramia longicauda*) | 13 | 31 | 0 | 44 | 16 | 37 |
| Blue-gray gnatcatcher (*Polioptila caerulea*) | 10 | 40 | 1 | 40 | 13 | 42 |
| Horned lark (*Eremophila alpestris*) | 8 | 48 | 2 | 33 | 13 | 43 |
| Western kingbird (*Tyrannus verticalis*) | 5 | 56 | 2 | 25 | 9 | 43 |
| * Other flycatchers | 10 | 38 | 0 | 44 | 13 | 45 |
| Tree swallow (*Tachycineta bicolor*) | 9 | 44 | 1 | 38 | 13 | 45 |
| Cliff swallow (*Petrochelidon pyrrhonota*) | 9 | 42 | 1 | 42 | 13 | 47 |
| Killdeer (*Charadrius vociferous*) | 7 | 55 | 2 | 30 | 12 | 48 |
| * Other longspurs | 9 | 43 | 0 | 44 | 12 | 49 |
| American robin (*Turdus migratorius*) | 8 | 49 | 1 | 41 | 11 | 50 |
| Ruby-throated hummingbird (*Archilochus colubris*) | 9 | 46 | 0 | 44 | 11 | 50 |
| Lesser nighthawk (*Chordeiles acutipennis)* | 9 | 47 | 0 | 44 | 11 | 52 |
| Chimney swift (*Chaetura pelagica*) | 8 | 50 | 0 | 43 | 11 | 53 |
| American goldfinch (*Spinus tristis*) | 8 | 51 | 0 | 44 | 10 | 54 |
| American pipit (*Anthus rubescens*) | 4 | 62 | 1 | 35 | 7 | 55 |
| House finch (*Haemorhous mexicanus*) | 7 | 53 | 0 | 44 | 9 | 55 |
| Savannah sparrow (*Passerculus sandwichensis*) | 7 | 54 | 0 | 44 | 9 | 57 |
| Yellow-rumped warbler (*Setophaga coronate*) | 5 | 56 | 0 | 44 | 6 | 58 |
| Cedar waxwing (*Bombycilla cedrorum*) | 5 | 58 | 0 | 44 | 6 | 59 |
| Lapland longspur (*Calcarius lapponicus*) | 5 | 58 | 0 | 44 | 6 | 59 |
| Yellow-billed cuckoo (*Coccyzus americanus*) | 5 | 60 | 0 | 44 | 6 | 61 |
| Baltimore oriole (*Icterus galbula*) | 5 | 61 | 0 | 44 | 6 | 62 |
| Indigo bunting (*Passerina cyanea*) | 3 | 63 | 0 | 44 | 4 | 63 |
| Common yellowthroat (*Geothlypis trichas*) | 3 | 64 | 0 | 44 | 4 | 64 |
| Ovenbird (*Seiurus aurocapillus*) | 0 | 65 | 0 | 44 | 0 | 65 |

The composite rank represents the sum of the percentage of strikes with damage and the percentage of strikes with substantial damage for that species group against all species. *denotes a species group. See S1 Table for a list of species in each species group (i.e. Other ducks). Strike data are from separate databases maintained by the USN (1990-2017) and USAF (1994-2017).
