# Supplementary material for: Quantification of avian hazards to military aircraft and implications for wildlife management
Source: PLoS One. 2018 Nov 1;13(11):e0206599. doi: 10.1371/journal.pone.0206599 (PMC6211720; doi:10.1371/journal.pone.0206599)
Supplement: S4 Table — (DOCX) [file pone.0206599.s004.docx]

**S4 Table. Relative hazard scores (RHS) for 65 species groups from most to least hazardous for cargo aircraft within the United States.**

| Species | % with damage | Damage rank | % with substantial damage | Substantial damage rank | Relative hazard score  (RHS) | Composite rank |
| --- | --- | --- | --- | --- | --- | --- |
| Snow goose (*Anser caerulescens*) | 83 | 1 | 45 | 1 | 100 | 1 |
| Canada goose (*Branta canadensis*) | 70 | 2 | 33 | 2 | 79 | 2 |
| Black vulture (*Coragyps atratus*) | 65 | 3 | 26 | 3 | 73 | 3 |
| Turkey vulture (*Cathartes aura*) | 63 | 4 | 25 | 4 | 68 | 4 |
| Swainson’s hawk (*Buteo swainsoni*) | 51 | 5 | 23 | 7 | 53 | 5 |
| Bald eagle (*Haliaeetus leucocephalus*) | 50 | 8 | 26 | 5 | 54 | 6 |
| Northern pintail (*Anas acuta*) | 51 | 7 | 18 | 10 | 51 | 7 |
| Mallard (*Anas platyrhynchos*) | 51 | 6 | 11 | 12 | 49 | 8 |
| Osprey (*Pandion haliaetus*) | 47 | 10 | 15 | 9 | 48 | 9 |
| Red-tailed hawk (*Buteo jamaicensis*) | 44 | 11 | 12 | 8 | 46 | 9 |
| Herring gull (*Larus argentatus*) | 38 | 14 | 16 | 6 | 44 | 11 |
| Double-crested cormorant (*Phalacrocorax auritus*) | 50 | 8 | 14 | 13 | 48 | 12 |
| Pied-billed grebe (*Podilymbus podiceps*) | 32 | 16 | 9 | 11 | 35 | 13 |
| * Other ducks | 40 | 12 | 8 | 16 | 39 | 14 |
| Great blue heron (*Ardea herodias*) | 39 | 13 | 10 | 19 | 37 | 15 |
| American coot (*Fulica americana*) | 32 | 15 | 8 | 21 | 30 | 16 |
| * Other falcons | 22 | 21 | 5 | 15 | 26 | 16 |
| Cattle egret (*Bubulcus ibis*) | 22 | 21 | 6 | 17 | 24 | 18 |
| * Other hawks | 27 | 18 | 8 | 20 | 26 | 18 |
| Great horned owl (*Bubo virginianus*) | 29 | 17 | 8 | 22 | 27 | 20 |
| Ring-billed gull (*Larus delawarensis*) | 21 | 25 | 5 | 18 | 22 | 21 |
| American crow (*Corvus brachyrhynchos*) | 15 | 33 | 4 | 14 | 21 | 22 |
| * Other gulls | 19 | 26 | 5 | 24 | 19 | 23 |
| Barn owl (*Tyto alba*) | 18 | 29 | 8 | 23 | 18 | 24 |
| Common grackle (*Quiscalus quiscula*) | 21 | 24 | 4 | 29 | 19 | 25 |
| White-throated swift (*Aeronautes saxatalis*) | 17 | 31 | 4 | 25 | 17 | 26 |
| Rock dove (*Columba livia*) | 16 | 32 | 4 | 26 | 16 | 27 |
| Yellow-billed cuckoo (*Coccyzus americanus*) | 19 | 28 | 3 | 32 | 16 | 28 |
| * Other doves | 15 | 33 | 3 | 28 | 15 | 29 |
| Baltimore oriole (*Icterus galbula*) | 12 | 37 | 2 | 27 | 12 | 30 |
| American robin (*Turdus migratorius*) | 12 | 35 | 2 | 34 | 11 | 31 |
| European starling (*Sturnus vulgaris*) | 11 | 39 | 4 | 30 | 11 | 31 |
| Yellow-bellied sapsucker (*Sphyrapicus varius*) | 10 | 43 | 2 | 31 | 10 | 33 |
| Cardinals, grosbeaks, and allies | 11 | 38 | 2 | 42 | 10 | 34 |
| * Other shorebirds | 10 | 45 | 1 | 36 | 9 | 35 |
| Mississippi kite (*Ictinia mississippiensis*) | 25 | 19 | 4 | 63 | 19 | 36 |
| Ovenbird (*Seiurus aurocapillus*) | 9 | 47 | 0 | 35 | 9 | 36 |
| Northern flicker (*Colaptes auratus*) | 24 | 20 | 2 | 63 | 19 | 38 |
| Brown thrasher (*Toxostoma rufum*) | 22 | 21 | 2 | 63 | 17 | 39 |
| Mourning dove (*Zenaida macroura*) | 9 | 49 | 3 | 38 | 8 | 40 |
| Great crested flycatcher (*Myiarchus crinitus*) | 7 | 55 | 2 | 33 | 7 | 41 |
| American kestrel (*Falco sparverius*) | 8 | 53 | 1 | 37 | 7 | 42 |
| White-winged dove (*Zenaida asiatica*) | 19 | 27 | 0 | 63 | 15 | 42 |
| * Other thrushes | 10 | 42 | 1 | 51 | 8 | 44 |
| Wood thrush (*Hylocichla mustelina*) | 18 | 30 | 0 | 63 | 14 | 44 |
| Purple martin (*Progne subis*) | 10 | 46 | 0 | 48 | 8 | 46 |
| Red-winged blackbird (*Agelaius phoeniceus*) | 8 | 52 | 1 | 45 | 7 | 47 |
| Ruby-crowned kinglet (*Regulus calendula*) | 7 | 57 | 2 | 41 | 6 | 48 |
| Lapland longspur (*Calcarius lapponicus*) | 6 | 60 | 0 | 39 | 6 | 49 |
| Short-eared owl (*Asio flammeus*) | 12 | 36 | 1 | 63 | 9 | 49 |
| Gray catbird (*Dumetella carolinensis*) | 10 | 44 | 1 | 57 | 8 | 51 |
| Killdeer (*Charadrius vociferous*) | 7 | 56 | 0 | 46 | 6 | 52 |
| Black-bellied plover (*Pluvialis squatarola*) | 11 | 40 | 2 | 63 | 8 | 53 |
| * Other plovers | 11 | 40 | 0 | 63 | 8 | 53 |
| * Meadowlarks | 6 | 64 | 0 | 40 | 6 | 55 |
| Indigo bunting (*Passerina cyanea*) | 6 | 63 | 0 | 43 | 6 | 56 |
| Sora (*Porzana carolina*) | 9 | 47 | 0 | 63 | 7 | 57 |
| American pipit (*Anthus rubescens*) | 6 | 68 | 0 | 44 | 5 | 58 |
| Northern mockingbird (*Mimus polyglottos*) | 9 | 50 | 0 | 63 | 7 | 59 |
| * Other flycatchers | 8 | 51 | 1 | 63 | 7 | 60 |
| American goldfinch (*Spinus tristis*) | 7 | 54 | 0 | 63 | 5 | 61 |
| Cedar waxwing (*Bombycilla cedrorum*) | 6 | 69 | 1 | 48 | 5 | 61 |
| Red-eyed vireo (*Vireo olivaceus*) | 6 | 61 | 1 | 57 | 5 | 63 |
| Common yellowthroat (*Geothlypis trichas*) | 5 | 70 | 1 | 50 | 5 | 64 |
| Brown-headed cowbird (*Molothrus ater*) | 6 | 58 | 0 | 63 | 5 | 65 |
| Golden-crowned kinglet (*Regulus satrapa*) | 6 | 59 | 0 | 63 | 5 | 66 |
| * Other wood warblers | 6 | 67 | 1 | 56 | 5 | 67 |
| Ruby-throated hummingbird (*Archilochus colubris*) | 6 | 61 | 1 | 63 | 5 | 68 |
| Dark-eyed junco (*Junco hyemalis*) | 5 | 72 | 1 | 53 | 4 | 69 |
| Common nighthawk (*Chordeiles minor*) | 4 | 76 | 1 | 52 | 4 | 70 |
| Savannah sparrow (*Passerculus sandwichensis*) | 4 | 81 | 0 | 47 | 3 | 70 |
| Scarlet tanager (*Piranga olivacea*) | 6 | 65 | 1 | 63 | 5 | 70 |
| Scissor-tailed flycatcher (*Tyrannus forficatus*) | 6 | 65 | 0 | 63 | 5 | 70 |
| Chimney swift (*Chaetura pelagica*) | 5 | 71 | 0 | 59 | 4 | 74 |
| Hermit thrush (*Catharus guttatus*) | 4 | 79 | 0 | 53 | 3 | 75 |
| Yellow-rumped Warbler (*Setophaga coronate*) | 5 | 73 | 1 | 61 | 4 | 76 |
| Bank swallow (*Riparia riparia*) | 4 | 77 | 1 | 60 | 3 | 77 |
| Bobolink (*Dolichonyx oryzivorus*) | 5 | 74 | 1 | 63 | 4 | 77 |
| Upland sandpiper (*Bartramia longicauda*) | 4 | 75 | 0 | 63 | 3 | 79 |
| Horned lark (*Eremophila alpestris*) | 3 | 85 | 0 | 55 | 3 | 80 |
| * Other sparrows | 4 | 78 | 1 | 62 | 3 | 80 |
| Cliff swallow (*Petrochelidon pyrrhonota*) | 4 | 80 | 1 | 63 | 3 | 82 |
| * Other vireos | 4 | 82 | 0 | 63 | 3 | 83 |
| Bank swallow (*Riparia riparia*) | 3 | 83 | 1 | 63 | 3 | 84 |
| Least sandpiper (*Calidris minutilla*) | 3 | 84 | 0 | 63 | 2 | 85 |
| Tree swallow (*Tachycineta bicolor*) | 3 | 86 | 0 | 63 | 2 | 86 |
| House wren (*Troglodytes aedon*) | 3 | 87 | 1 | 63 | 2 | 87 |
| Western kingbird (*Tyrannus verticalis*) | 3 | 88 | 0 | 63 | 2 | 88 |
| * Other wrens | 3 | 89 | 0 | 63 | 2 | 89 |
| Wilson's snipe (*Gallinago delicate*) | 2 | 90 | 0 | 63 | 2 | 90 |
| Common snipe (*Gallinago gallinago*) | 2 | 91 | 0 | 63 | 2 | 91 |
| Blue-gray gnatcatcher (*Polioptila caerulea*) | 0 | 92 | 0 | 63 | 0 | 92 |
| Burrowing owl (*Athene cunicularia*) | 0 | 92 | 0 | 63 | 0 | 92 |
| Dunlin (*Calidris alpine*) | 0 | 92 | 0 | 63 | 0 | 92 |
| House finch (*Haemorhous mexicanus*) | 0 | 92 | 0 | 63 | 0 | 92 |
| Lesser nighthawk (*Chordeiles acutipennis)* | 0 | 92 | 0 | 63 | 0 | 92 |
| * Other longspurs | 0 | 92 | 0 | 63 | 0 | 92 |
| Snow bunting (*Plectrophenax nivalis*) | 0 | 92 | 0 | 63 | 0 | 92 |

The composite rank represents the sum of the percentage of strikes with damage and the percentage of strikes with substantial damage for that species group against all species. * denotes a species group. See S1 Table for a list of species in each species group (i.e. Other ducks). Strike data are from separate databases maintained by the USN (1990-2017) and USAF (1994-2017).
