# Supplementary material for: Quantification of avian hazards to military aircraft and implications for wildlife management
Source: PLoS One. 2018 Nov 1;13(11):e0206599. doi: 10.1371/journal.pone.0206599 (PMC6211720; doi:10.1371/journal.pone.0206599)
Supplement: S5 Table — (DOCX) [file pone.0206599.s005.docx]

**S5 Table. Relative hazard scores (RHS) for 12 species groups from most to least hazardous for stealth aircraft within the United States.**

| Species | % with damage | Damage rank | % with substantial damage | Substantial damage rank | Relative hazard score  (RHS) | Composite rank |
| --- | --- | --- | --- | --- | --- | --- |
| American robin (*Turdus migratorius*) | 12 | 2 | 4 | 1 | 85 | 1 |
| Mourning dove (*Zenaida macroura*) | 16 | 1 | 2 | 2 | 100 | 1 |
| Cliff swallow (*Petrochelidon pyrrhonota*) | 10 | 3 | 0 | 5 | 56 | 3 |
| Common nighthawk (*Chordeiles minor*) | 9 | 4 | 0 | 5 | 48 | 4 |
| Savannah sparrow (*Passerculus sandwichensis*) | 4 | 5 | 0 | 5 | 24 | 5 |
| American kestrel (*Falco sparverius*) | 4 | 6 | 0 | 5 | 22 | 6 |
| Barn swallow (*Hirundo rustica*) | 3 | 7 | 0 | 5 | 18 | 7 |
| * Other wood warblers | 3 | 7 | 0 | 5 | 18 | 7 |
| Horned lark (*Eremophila alpestris*) | 3 | 10 | 1 | 3 | 22 | 9 |
| * Meadowlarks | 3 | 9 | 0 | 5 | 17 | 10 |
| Killdeer (*Charadrius vociferous*) | 2 | 11 | 0 | 5 | 12 | 11 |
| * Other sparrows | 2 | 12 | 1 | 4 | 15 | 11 |

The composite rank represents the sum of the percentage of strikes with damage and the percentage of strikes with substantial damage for that species group against all species. * denotes a species group. See S1 Table for a list of species in each species group (i.e. Other ducks). Strike data are from separate databases maintained by the USN (1990-2017) and USAF (1994-2017).
