# Supplementary material for: Quantification of avian hazards to military aircraft and implications for wildlife management
Source: PLoS One. 2018 Nov 1;13(11):e0206599. doi: 10.1371/journal.pone.0206599 (PMC6211720; doi:10.1371/journal.pone.0206599)
Supplement: S6 Table — (DOCX) [file pone.0206599.s006.docx]

**S6 Table. Relative hazard scores (RHS) for 16 species groups from most to least hazardous for stealth aircraft within the United States.**

| Species | % with damage | Damage rank | % with substantial damage | Substantial damage rank | Relative hazard score  (RHS) | Composite rank |
| --- | --- | --- | --- | --- | --- | --- |
| Mourning dove (*Zenaida macroura*) | 22 | 2 | 9 | 1 | 85 | 1 |
| Turkey vulture (*Cathartes aura*) | 28 | 1 | 8 | 2 | 100 | 1 |
| * Other thrushes | 9 | 3 | 0 | 5 | 25 | 3 |
| Cliff swallow (*Petrochelidon pyrrhonota*) | 8 | 4 | 0 | 5 | 23 | 4 |
| * Other gulls | 7 | 5 | 0 | 5 | 21 | 5 |
| Other sparrows | 7 | 6 | 1 | 4 | 23 | 5 |
| Yellow-billed cuckoo (*Coccyzus americanus*) | 5 | 8 | 5 | 3 | 26 | 7 |
| Gray catbird (*Dumetella carolinensis*) | 6 | 7 | 0 | 5 | 16 | 8 |
| Rock dove (*Columba livia*) | 4 | 9 | 0 | 5 | 12 | 9 |
| Yellow-rumped warbler (*Setophaga coronate*) | 4 | 10 | 0 | 5 | 10 | 10 |
| Red-eyed vireo (*Vireo olivaceus*) | 3 | 11 | 0 | 5 | 8 | 11 |
| Horned lark (*Eremophila alpestris*) | 3 | 12 | 0 | 5 | 8 | 12 |
| * Other wood warblers | 2 | 13 | 0 | 5 | 5 | 13 |
| Chimney swift (*Chaetura pelagica*) | 2 | 14 | 0 | 5 | 4 | 14 |
| Barn swallow (*Hirundo rustica*) | 0 | 15 | 0 | 5 | 0 | 15 |
| Common yellowthroat (*Geothlypis trichas*) | 0 | 15 | 0 | 5 | 0 | 15 |

The composite rank represents the sum of the percentage of strikes with damage and the percentage of strikes with substantial damage for that species group against all species. * denotes a species group. See S1 Table for a list of species in each species group (i.e. Other ducks). Strike data are from separate databases maintained by the USN (1990-2017) and USAF (1994-2017).
