# Supplementary material for: Quantification of avian hazards to military aircraft and implications for wildlife management
Source: PLoS One. 2018 Nov 1;13(11):e0206599. doi: 10.1371/journal.pone.0206599 (PMC6211720; doi:10.1371/journal.pone.0206599)
Supplement: S7 Table — No significant relationship was found for stealth airframes and avian log body mass. Bold values indicate best fit by the coefficient of determination (r2 values). (DOCX) [file pone.0206599.s007.docx]

**S7 Table. Summary of quadratic and linear relationships with military airframe and avian log body mass. No significant relationship was found for stealth airframes and avian log body mass. Bold values indicate best fit by the coefficient of determination (*r^2^* values).**

| Airframe | Linear relationship *p* value | Linear relationship fit | Quadratic relationship *p* value | Quadratic relationship fit |
| --- | --- | --- | --- | --- |
| Cargo | < 0.00 | 0.68 | < 0.00 | **0.82** |
| Fighter | < 0.00 | 0.68 | < 0.00 | **0.78** |
| Rotorcraft | 0.003 | **0.44** | 0.01 | 0.40 |
| Stealth | 0.26 | 0.04 | 0.53 | -0.06 |
| All airframes | < 0.00 | 0.63 | < 0.00 | **0.76** |
