# Supplementary material for: Quantification of avian hazards to military aircraft and implications for wildlife management
Source: PLoS One. 2018 Nov 1;13(11):e0206599. doi: 10.1371/journal.pone.0206599 (PMC6211720; doi:10.1371/journal.pone.0206599)
Supplement: S8 Table — Results from the binomial generalized linear model of factors that influence the probability of a bird strike to cause (a) any damage and the probability of a bird strike causing (b) substantial damage with military aircraft. (DOCX) [file pone.0206599.s008.docx]

**S8 Table. Results from the binomial generalized linear model of factors that influence the probability of a bird strike to cause (a) any damage and the probability of a bird strike causing (b) substantial damage with military aircraft.**

| **(a) Damage model** | **Residual df** | **Residual Deviance** | **k** | **AICc** | **ΔAICc** | **w*_i_*** |  |
| --- | --- | --- | --- | --- | --- | --- | --- |
| Flyway + airframe + log mass + reporting branch + airframe x log mass | 32302 | 20216 | 12 | 20240 | 0.00 | 1.00 |  |
| Flyway + airframe + log mass + reporting branch + flyway x log mass | 32302 | 20280 | 12 | 20304 | 64.0 | 0.00 |  |
| Flyway + airframe + log mass + reporting branch + flyway x airframe | 32296 | 20275 | 18 | 20310 | 70.8 | 0.00 |  |
| Flyway + airframe + log mass + reporting branch | 32305 | 20323 | 9 | 20341 | 101.2 | 0.00 |  |
| **(b) Substantial damage model** | **Residual df** | **Residual Deviance** | **k** | **AICc** | **ΔAICc** | **w*_i_*** |  |
| Flyway + airframe + log mass + reporting branch + airframe x log mass | 32302 | 6386 | 12 | 6409 | 0.00 | 1.00 |  |
| Flyway + airframe + log mass + reporting branch + flyway x log mass | 32302 | 6415 | 12 | 6439 | 38.7 | 0.00 |  |
| Flyway + airframe + log mass + reporting branch + flyway x airframe | 32296 | 6405 | 18 | 6441 | 31.2 | 0.00 |  |
| Flyway + airframe + log mass + reporting branch | 32305 | 6428 | 9 | 6446 | 36.3 | 0.00 |  |
